# Active surveillance of prostate cancer: a questionnaire survey of urologists, clinical oncologists and urology nurse specialists across three cancer networks in the United Kingdom

**DOI:** 10.1186/s12894-015-0049-y

**Published:** 2015-06-13

**Authors:** Yiannis Philippou, Hary Raja, Vincent J. Gnanapragasam

**Affiliations:** Department of Surgery, Basildon & Thurrock University Hospital, Essex, SS16 5NL UK; Academic Urology Group, Department of Surgery & Oncology, University of Cambridge, Cambridge Biomedical Campus, Cambridge, CB2 0QQ UK

**Keywords:** Prostate cancer, Active surveillance, Questionnaire survey

## Abstract

**Background:**

Active surveillance is considered a mainstream strategy in the management of patients with low-risk prostate cancer. A mission-critical step in implementing a robust active surveillance program and plan its resource and service requirements, is to gauge its current practice across the United Kingdom. Furthermore it is imperative to determine the existing practices in the context of the recommendations suggested by the recent National Institute for Health and Clinical Excellence guidance on active surveillance of prostate cancer.

**Methods:**

An internet questionnaire was circulated to urologists, clinical oncologists and urology nurse specialists across three geographically distinct cancer networks. Twenty five questions across four domains were assessed. (i) hospital resources (staff and clinical areas) utilised for active surveillance (ii) enrolment criteria (iii) follow up (iv) criteria that trigger conversion to active treatment.

**Results:**

We received 35 responses, 20 of which were from urologists. The survey data suggests that there is marked heterogeneity in enrolment criteria with patients having features of intermediate-risk prostate cancer often recruited into Active Surveillance programs. Only 60 % of our respondents use multiparametric MRI routinely to assess patient suitability for active surveillance. In addition, marked variation exists in how patients are followed up with regard to PSA testing intervals and timing of repeat biopsies. Only 40 % undertake a repeat biopsy at 12 months. Tumour upgrading on repeat biopsy, an increase in tumour volume or percentage of core biopsies involved would prompt a recommendation for treatment amongst most survey respondents. In addition allocation of resources and services for active surveillance is poor. Currently there are no dedicated active surveillance clinics, which are well-structured, -resourced and -supported for regular patient counselling and follow up.

**Conclusion:**

This variability in enrolment criteria and follow up is also demonstrated in international and national series of active surveillance. Resources are not currently in place across the UK to support an active surveillance program and a national discussion and debate to plan resources is much required so that it can become a mainstream therapeutic strategy.

**Electronic supplementary material:**

The online version of this article (doi:10.1186/s12894-015-0049-y) contains supplementary material, which is available to authorized users.

## Background

There is a clear trend, both in the UK and worldwide, towards managing patients diagnosed as having low-risk prostate cancer (LRPC) with active surveillance (AS) [1, 2]. The recent publication of the Prostate Cancer Intervention Versus Observation Trial (PIVOT) has further added to the growing evidence which supports that LRPC can be safely managed by AS and without the treatment-related side effects [3]. There are however major caveats in considering widespread adoption of AS for LRPC in the UK and indeed in other health-care systems. Firstly, there is an increasing concern that the current diagnostic method of an elevated prostate specific antigen (PSA), digital rectal examination (DRE) and a single 10–12 core trans-rectal biopsy, carries a significant potential of missing higher risk disease. Secondly there remain no universally accepted inclusion criteria for AS and there is a lack of consensus on what an AS regime should consist of. Whereas there are national and international guidelines on standards of surgery and radiotherapy for prostate cancer these are lacking for AS [4]. Thirdly, the timing of PSA checks, repeat examination, place and role of imaging, repeat biopsies and triggers for intervention vary considerably from centre to centre and even from clinician to clinician. Finally, AS requires structured, well-resourced and supported clinics for regular patient reviews, and these will be needed for many years. It is therefore clear that an expansion of AS will be a significant resource implication for any health service let alone an already overstretched National Health Service (NHS).

A move to a wider implementation of AS will require in addition to a uniform protocol, a national consensus on the resource and service requirements in setting this up and on the likely cost-implications of a long-term AS programme. As an initial step in this process the recent publication of the updated National Institute for Health and Clinical Excellence (NICE) guidance (CG175) has proposed a guideline for how men on AS may be managed (Table 1) [5]. A mission-critical step however in any attempt to adopt this guideline, is to gauge how AS is currently practiced in the UK.

**Table 1:** Protocol for Active Surveillance as outlined by NICE: prostate cancer: diagnosis and treatment (CG175)

Timing	Tests
At enrolment in active surveillance	Multiparametric MRI if not previously performed
Year 1 of active surveillance	Every 3–4 months: measure PSA
	Throughout active surveillance: monitor PSA kinetics
	Every 6–12 months: DRE
	At 12 months prostate rebiopsy
Years 2–4 of active surveillance	Every 3–6 months: measure PSA
	Throughout active surveillance: monitor PSA kinetics
	Every 6–12 months: DRE
Year 5 and every year thereafter until active surveillance ends	Every 6 months: measure PSA
	Throughout active surveillance: monitor PSA kinetics
	Every 12 months: DRE

## Methods

We conducted an online survey of urologists, clinical oncologists (these were both medical and radiation oncologists with a special interest in uro-oncology) and urology nurse specialists with regard to the practice of AS within the East of England (EoE) cancer network. Data was collected during the year 2012–2013. The EoE cancer network delivers cancer care to 2.63 million people and is comprised mainly of two university hospitals and six district general hospitals. It employs approximately 50 consultant urologists. An internet questionnaire was circulated by email to urological departments of the eight hospitals within the network. The email address of each urological department within each hospital was available on the hospital website. After receiving the email secretarial staff within each hospital were able to forward the questionnaire to all urological consultants, oncologists and urology nurse specialists working within each hospital. A further email was sent 1 month after in order to remind non-responders to complete the questionnaire. We also distributed the survey to two other cancer networks in geographically distinct areas of the UK. These were the North of England Cancer network and the Avon Somerset and Wiltshire cancer network. The reason behind the inclusion of a further two geographically distinct cancer networks was that we felt that this would allow us to form a more comprehensive opinion on the practice of AS across the UK and also allow comparison of our practice with other networks. Twenty five questions across four domains were assessed with associated multiple-choice answers (Additional file 1). Where more than one answer was possible respondents were able to select more options. The four domains were: (i) what hospital resources are currently utilised (staff and clinical areas) to counsel and follow up patients on AS (ii) enrolment criteria for AS (iii) how patients on AS are followed up (iv) respondents opinions on criteria that would trigger conversion to active treatment. Results were collated and rounded up to the closest percentage and represent the frequency of the answer selected for a particular question against the number of respondents answering the question.

### Ethics approval

The above study is registered as an audit at Addenbrooke’s hospital NHS trust. Ref: 3631.

## Results

Completed questionnaires were received from 15 urologists, six clinical oncologists and four specialist nurses within our cancer network with all invited trusts taking part. We received a further 10 responses from the other two cancer networks we surveyed (Table 2). From the 35 respondents, 31 were directly involved in managing patients on AS with most centres reported managing more than 30 men a year by AS.

**Table 2:** Distribution of responses according to specialty

Specialty	Response percent (%)	Response count
Urology	57	20
Medical oncology	0	0
Clinical oncology	20	7
Urology specialist nurse	20	7
Oncology specialist nurse	3	1
Total		35
Skipped question		0

### Current setting of AS

**Fig. 1:**
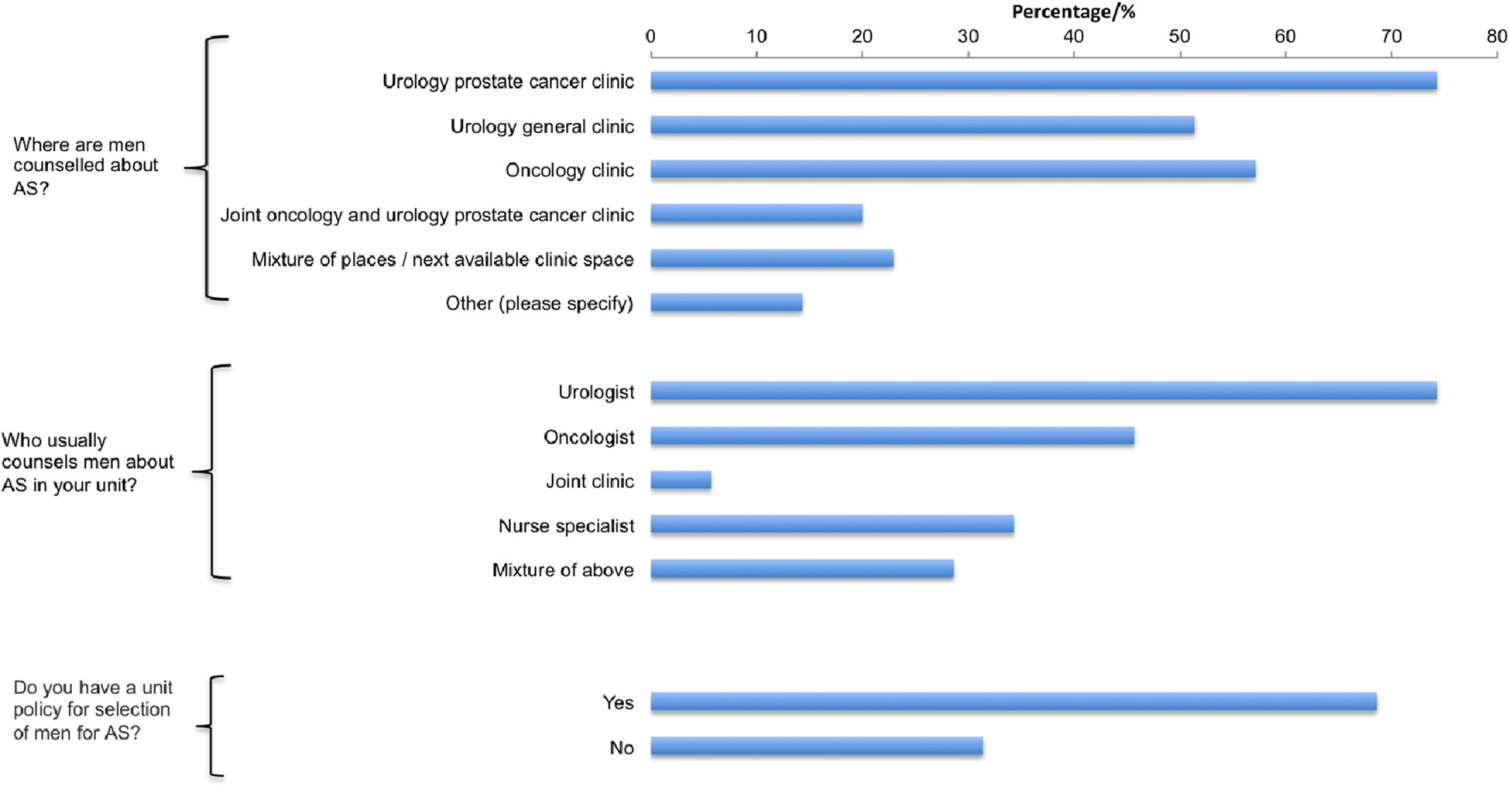
Responses on the resources currently utilised to counsel men on AS. The percentage represents the frequency of the answer selected for a particular question against the number of respondents answering the question

Resources allocated to AS counselling were first assessed (Fig. 1). Dedicated urology prostate cancer clinics were used in 74 % of cases but counselling and reviews also occurred in general urology clinics, oncology clinics, joint oncology and urology clinics as well as nurse led clinics. AS counselling was primarily done by urologists in 75 % of cases. However oncologists and nurse specialists were also actively involved in this process. Respondents were then asked about the existence of an AS policy used in their unit. Of all respondents 68 % reported the use of a policy to guide selection of men suitable for AS. The rest however stated of not being aware of any agreed policy. These results suggest that currently there is little evidence of a dedicated service for men managed by AS within current NHS resource provisions.

### Enrolment criteria for AS

**Fig. 2:**
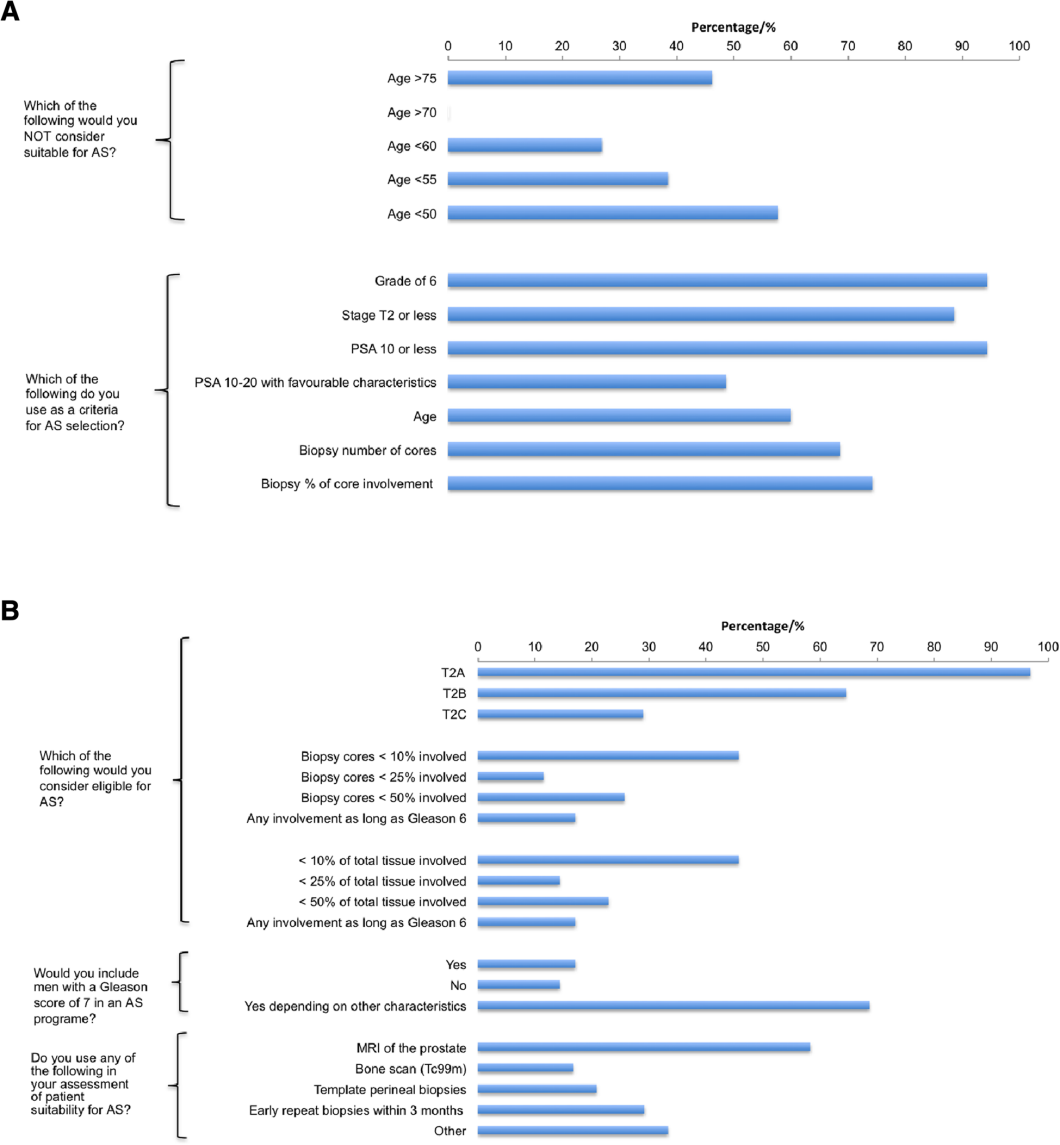
**a** Opinions on enrolment criteria for AS. **b** Opinions on enrolment criteria for AS

Respondents were then asked about different enrolment criteria used in enlisting patients to their AS programme (Fig. 2a). With regards to age, there was a strong agreement that AS would not be considered in men of 55 years or younger. A sizeable proportion (46 %) would not consider it in men over 75 years either with the preference here being for watchful waiting. There was a strong agreement also that a classical definition of low risk (Gleason score of 6, a PSA level of ≤10 ng/ml and a TNM stage of ≤ T2) were necessary for enrolment into an AS programme. Some would also consider a PSA level between 10 and 20 ng/ml as long as other characteristics such as Gleason score and TNM stage were favourable. 64.5 % and 29 % of respondents would consider patients for AS if the TNM stage was T2b or T2c respectively. A significant majority considered the number of cores involved as well as the percentage of the core involved as important points in decision making for AS enrolment. Specifically, 17 % would not be deterred from AS if more than 50 % of total number of cores biopsied were involved as long as the Gleason score is 6. We also observed that patients were being enrolled in programs with characteristics of intermediate risk prostate cancer (Fig. 2b). Interestingly, nearly 70 % would consider AS in patients with a Gleason score of 7 if other tumour characteristics such as TNM stage, age, PSA level and information on biopsy core involvement were favourable. 58.3 % routinely use multiparametric MRI (mp MRI) and 29.2 % perform an early repeat biopsy (TRUS or template) within 3 months to aid in the selection of patients suitable for AS. These findings highlight the current significant variability and lack of standardisation in the inclusion criteria for men on AS across hospitals even within a single network.

### Follow up of patients on AS

**Fig. 3:**
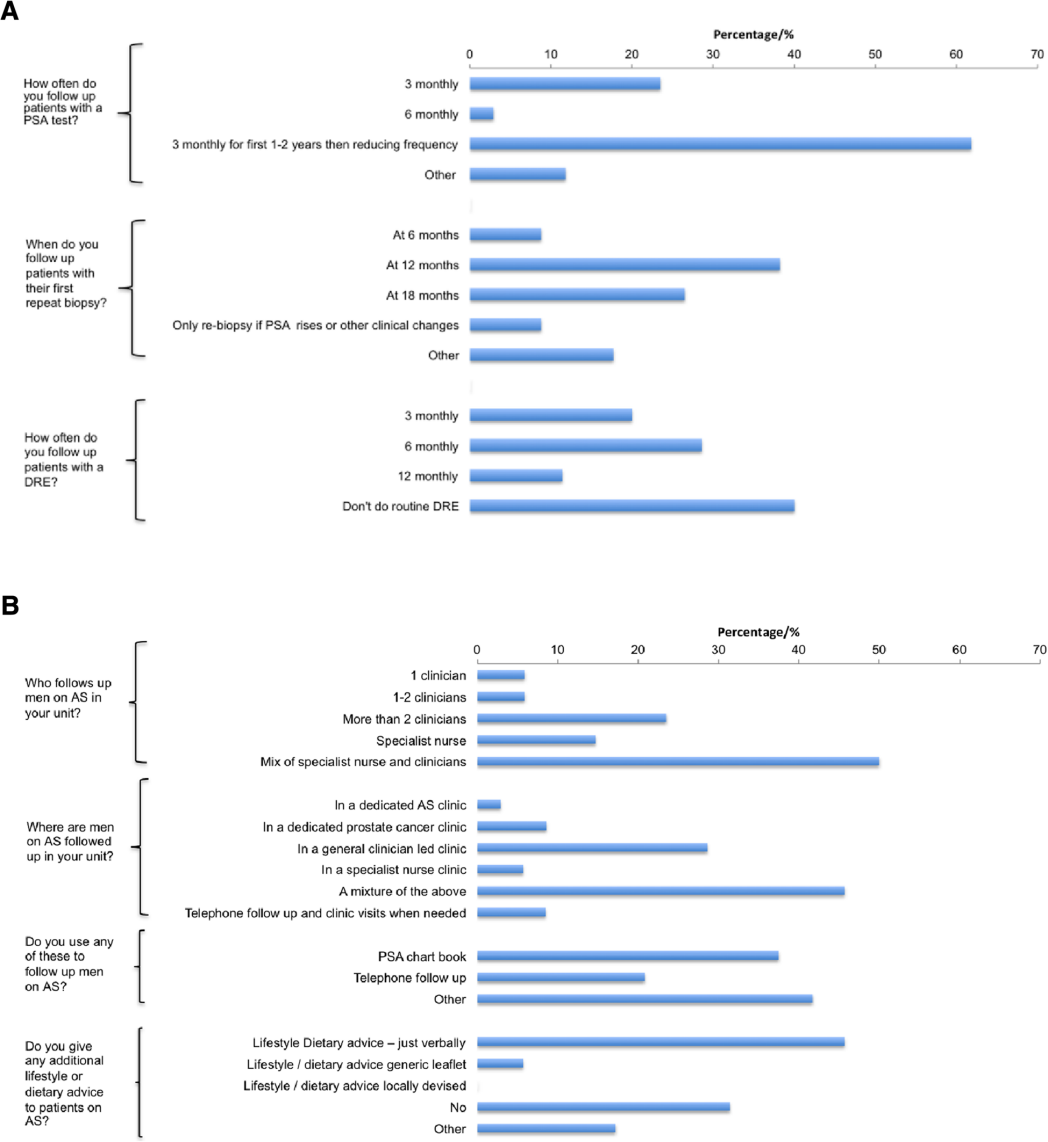
**a** Responses on how men on AS are currently followed up. **b** Responses on resources currently utilised to follow up men on AS

**Fig. 4:**
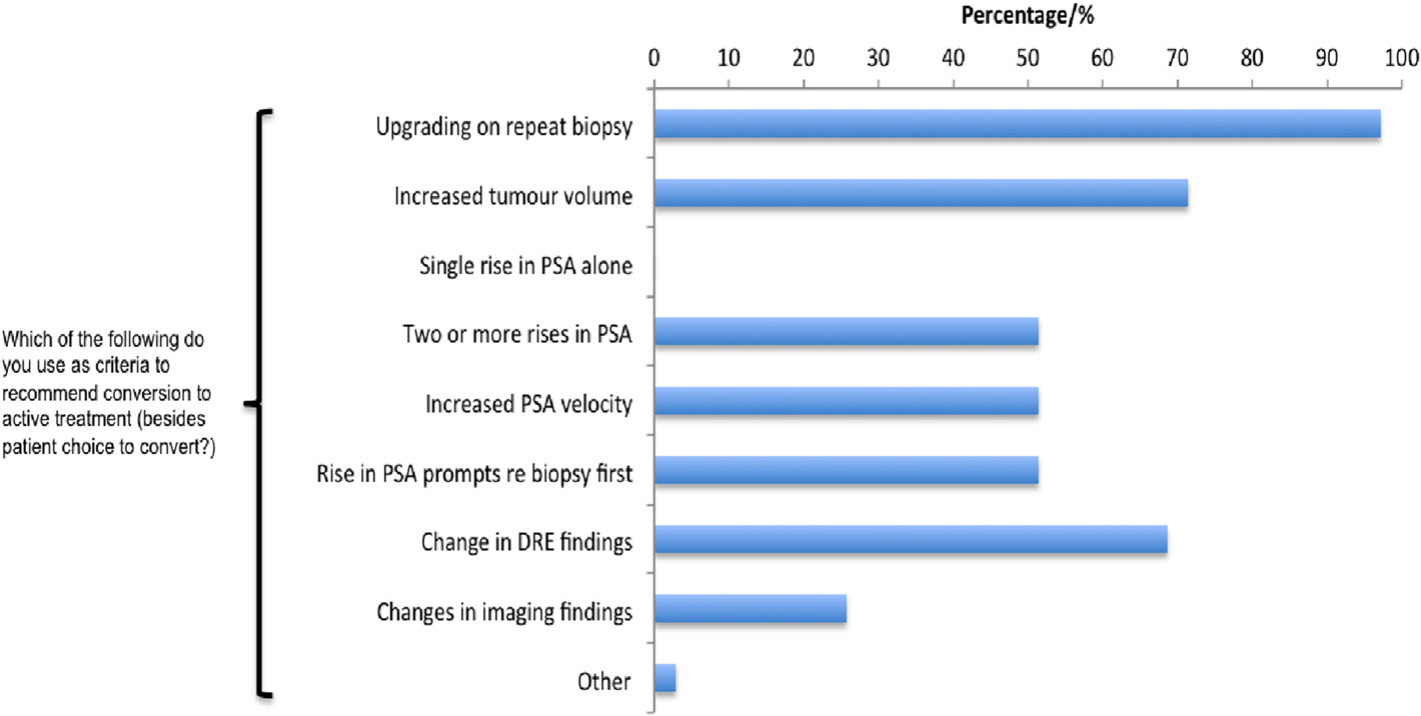
Respondents views on criteria that would trigger conversion to active treatment

Respondents were asked about how they followed up patients on AS (Fig. 3a). The majority of respondents (62 %) indicated that they followed patients up with 3 monthly PSA at least in the first 2 years on AS and nearly 40 % would carry out the first repeat biopsy within 12 months. A significant minority however would only re-biopsy if there was evidence of change in the serum PSA or other clinical changes. Most respondents (60 %) used DRE as an integral part of AS monitoring. The vast majority did not use MRI as a tool to monitor men on AS. Follow up was undertaken in a multidisciplinary setting with at least two clinicians or a mixture of clinicians and nurse specialists involved in patient care (Fig. 3b). This however did not often occur in dedicated AS prostate cancer clinics but mainly in clinician led general urology clinics. There was also a mixture of follow up methods including PSA chart books and telephone checks. A majority of respondents also stated that they felt it was important to give patients on AS certain life-style and dietary advice either verbally or in the form of a patient information leaflet. Finally, we asked what would trigger a recommendation for active treatment. Here there was broad agreement that tumour upgrading on repeat biopsy or an increase in tumour volume or percentage of core biopsies involved would prompt a recommendation for treatment. Half of our respondents however would recommend active treatment based on evidence of a rising PSA alone or an increase in PSA velocity. Finally changes in DRE findings as well as evidence of radiological progression of the tumour would prompt radical treatment in more than 50 % of cases (Fig. 4).

### Comparison with other network trusts

**Fig. 5:**
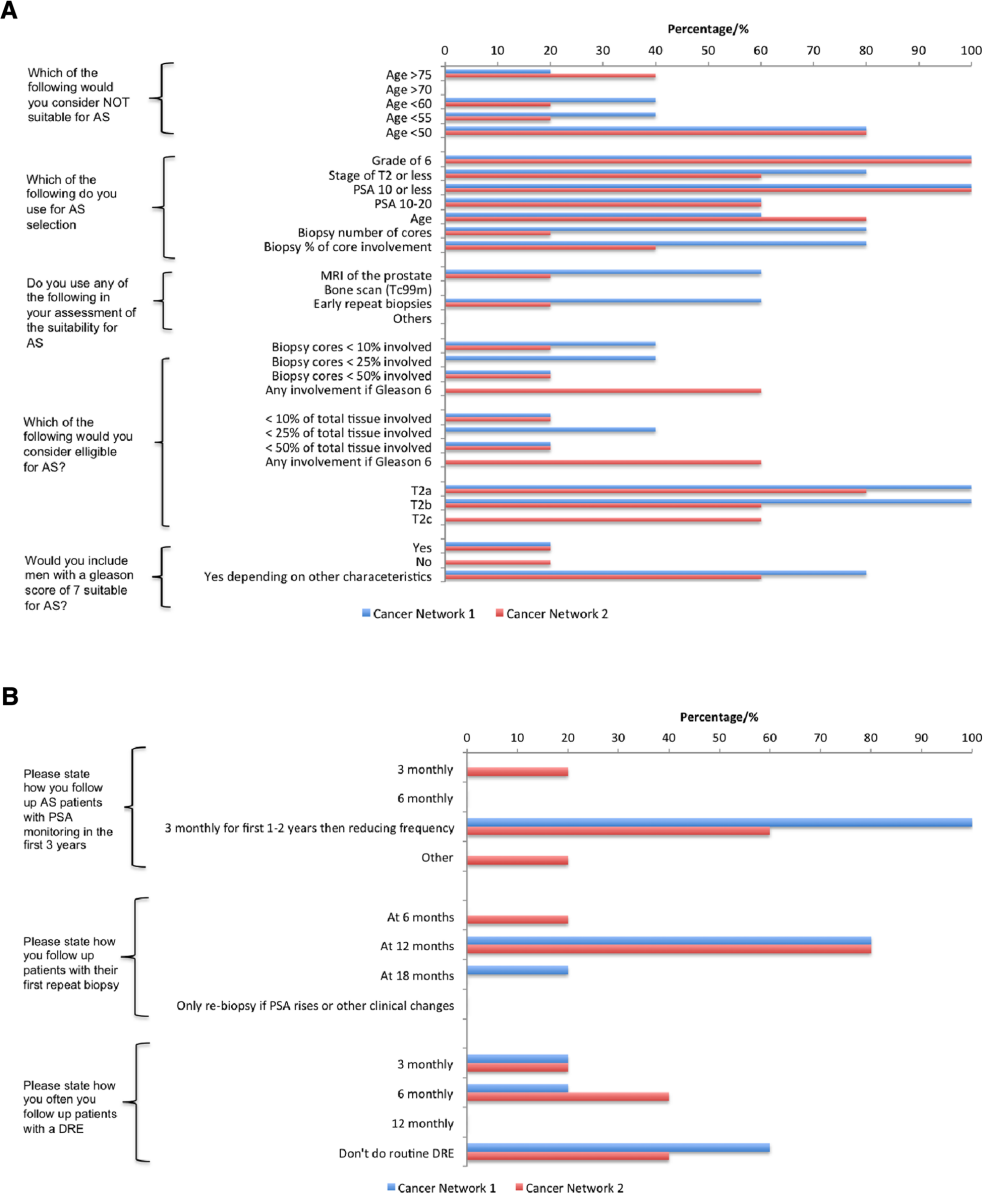
**a** Respondents views on enrolment criteria for AS from the two geographically distinct cancer networks surveyed. **b** Respondents views on how they follow up patients on AS from the two geographically distinct cancer networks surveyed

The same questionnaire was sent out to members of trusts part of two other UK cancer networks. We received a total of 10 responses, five from each cancer network. The findings were very comparable to our own network. There was broad agreement that men under 50 years would not be suitable for AS. In both networks surveyed, there was significant variation in respondent’s views on inclusion criteria for AS (Fig. 5a). Similar to our data, most respondents would include Gleason grade 7 patients in an active surveillance programme but dependent on other clinical characteristics. In one of the two networks studied, a significant number of respondents advocated MRI and transperineal repeat biopsies as part of their assessment of patients suitability for AS. However only a minority of respondents in the other network used any additional biopsy or imaging in evaluating patients suitability. On the question of timing of repeat biopsies there was again variability in the responses (Fig. 5b). The majority advocated re-biopsy at 12 or 18 months after entry onto an AS programme. Finally, we compared triggers to initiate a change in management. Here there was broad consistency in terms of what would initiate a change and included an increase in grade and/or tumour volume. This comparison demonstrated that the lack of dedicated resources and variability in inclusion and follow up in AS is likely to be a universal issue across the NHS.

## Discussion

In contemporary UK practice LRPC accounts for 20 % of all new prostate cancer diagnoses [6]. The perception that LRPC is over-treated has gained the ascendancy among the urological community following results from randomised studies such as the European Randomised Study of Screening for Prostate Cancer (ERSPC) trial [7] and Prostate Cancer Intervention vs Observation Trial (PIVOT) [8]. It is therefore easy to foresee that AS will most likely become the preferred option of managing patients with LRPC. The UK NICE guidelines define men suitable for AS as having the following characteristics: clinical stage T1c; a Gleason score of 3 + 3; a PSA density of < 0.15 ng/mL/mL; and cancer in < 50 % of their total number of biopsy cores with < 10 mm of any core involved (http://guidance.nice.org.uk/CG175) [5]. These recommendations are very similar to the European, American and Canadian urological guidelines which universally recommend that AS is suitable for patients with Gleason score of 6 or less [1, 2, 9]. Our survey demonstrated that the NICE guidelines regarding AS enrolment are generally followed. However, in certain cases patients are being recruited into AS programmes with characteristics of intermediate-risk prostate cancer. A significant proportion of our respondents considered men for AS who had T2b or T2c disease, a PSA of 10–20 ng/ml, a Gleason score of 7 and patients with tumour involvement of more than 50 % of their total biopsy cores. This reflects that routine UK practice of AS commonly outstretches that described in most international published series of AS and also the recommendations of European, American and Canadian urological guidelines. Encouragingly however, there was a general agreement between our respondents as to the criteria that would trigger conversion to radical treatment.

Our survey demonstrated the marked heterogeneity which exists in the practice of AS across our cancer network. This variability is not unique to our cancer network alone but also evident in other cancer networks too in geographically distinct areas of the UK. This striking variability in AS enrolment criteria, follow up and triggers for intervention is also demonstrated in international and national series of AS (Tables 3 and 4). In these studies, patients were followed up with a combination of repeat biopsies, serial PSA measurements and clinical examination. The frequency of repeat biopsies varied widely analogous to our own cancer network. Some carried out biopsies annually [10–12], while others every 2 or 3 years [13–16], and in some depending on clinical characteristics [17, 18]. Almost unanimously in the studies we reviewed tumour upgrading on repeat biopsy would prompt a recommendation for treatment. Some studies also considered an increase in tumour volume or percentage of core biopsies involved as a trigger to radical treatment. PSA doubling time or velocity was also sometimes a trigger to proceed to radical treatment.

**Table 3:** Selection criteria for Active Surveillance in international published series

Publication	Gleason Score	PSA (ng/ml)	Positive cores	% positive biopsy cores	% cancer involvement per core	cT
Dall’ Era [11]	≤6	<10	-	<33	-	≤2a
Bul [10]	≤7	<20	≤3	-	-	-
Soloway [12]	≤6	≤10	≤2	-	<20	≤2
Tosoian [18]	≤6	≤10	≤2	-	<50	1c
Ercole [13]	≤6	<10	≤2	-	<50	≤2a
Klotz [14]	≤6	≤10	-	-	-	≤2b
Ischia [15]	≤6	<10	-	-	-	≤2a
Thomsen [16]	≤6	≤10	≤3	-	<50	≤2a
Selvadurai [17]	≤6	<15	-	≤50	-	≤2

*cT* clinical tumour category, *PSA* prostate specific antigen

**Table 4:** Triggers to treatment used in patients under Active Surveillance

Publication	Gleason Score on repeat biopsy	Positive cores	% cancer involvement per single core	% positive biopsy cores	PSAdt cT (years)	PSAv (ng/ml/year)	cT
Dall’Era [11]	Progression	-	-	-	-	>0.75	-
Tosoian [18]	>6	>2	>50	-	-	-	-
Ercole [13]	Progression	Increase	Increase	-	-	-	Upstage
Klotz [14]	≥4	-	-	-	3	-	-
Ischia [15]	Progression	-	-	-	-	-	Upstage
Thomsen [16]	≥3 + 4	>3	-	-	3	-	Upstage
Selvadurai [17]	≥4 + 3	-	-	>50	-	>1	-

*cT* clinical tumour category, *PSAdt* prostate-specific antigen doubling time, *PSAv* PSA velocity

The updated NICE guidance has advocated the use of mpMRI at the time of AS enrolment followed by a repeat biopsy at year one and has put in place a follow up regime as its key suggestions. To understand what the impact of these recommendations would be on routine clinical practice, our survey assessed the current reported patterns of practice. Amongst our respondents only 40 % of respondents performed a repeat biopsy at 12 months and only 60 % use mpMRI routinely as a tool for selecting patients suitable for AS. It should be noted however that although the NICE guidelines have clearly stated that mpMRI should occur at the time of AS enrolment, the EUA and AUA guidelines are not so prescriptive. Similarly Canadian urological guidelines from Ontario recommend that mpMRI may be included in AS protocols but is not currently a necessity [1, 2, 9]. In contrast, the recommendation on 3–4 monthly PSA checks is consistent with current routine practice in our survey. Even so there will need to be a method to monitor and track the PSA. Similarly recommendations for DRE every 6–12 months is probably best combined with clinic reviews. Of note, in our survey there was great variability in who did the AS follow up including DRE. Moreover 40 % of respondents did not routinely perform a DRE during the follow up appointment. Again adherence to the NICE guidelines will require a significant change in practice and ideally necessitate consistency in AS follow up providers.

From the results of this survey it is quite clear that the implementation of a robust and consistent AS program according to the recommendations of the NICE guidelines will prove challenging due to the current variation in its practice. In addition the burden of follow-up with clinical examinations and serum PSA testing on both men and healthcare systems is far from cost-neutral. Even more so the use of novel strategies such as mpMRI at the time of AS enrollment will further put a strain on radiology providers. It is clear that in addition to reducing the variability in the practice of AS there is also the need for robust cost-effectiveness studies to ensure that such novel strategies are both clinically and cost-effective.

This study however does not come without its limitations. Firstly the methodology of this study is a questionnaire survey study with a total of 35 respondents which is a low number of participants and may not accurately depict the practice of AS within the EoE cancer network. Also this study was UK specific and may not mirror the trends in the world-wide practice of AS though our review of other international published series of AS did demonstrate variability in its practice. Furthermore the majority of our respondents were from primarily academic institutions therefore it is likely that the practice of AS is even more heterogeneous outside the academic setting.

## Conclusions

Despite its limitations, the present survey has demonstrated the marked heterogeneity in which AS is practiced across a cancer network in the UK. This is also mirrored across other hospitals in the UK. It is clear that if the NICE guidelines are adopted then the resource requirements are likely to be very significant and not currently in place across the UK. These issues need urgent resolution and now is the time for a national discussion and planning. This would provide the much needed reassurance to clinicians and patients of the robustness of an AS programme on a par with current standard therapeutic options.

## Additional file

Additional file 1:
**Questions included in survey.**

## Abbreviations

LRPC: Low-risk prostate cancer
AS: Active Surveillance
EoE: East of England
PIVOT: Prostate Cancer Intervention Versus Observation Trial
PSA: Prostate specific antigen
DRE: Digital Rectal Examination
NICE: National Institute for Health and Clinical Excellence
NHS: National Health Service
mp MRI: Multiparametric MRI
