# Supplementary material for: Active surveillance of prostate cancer: a questionnaire survey of urologists, clinical oncologists and urology nurse specialists across three cancer networks in the United Kingdom
Source: BMC Urol. 2015 Jun 13;15:52. doi: 10.1186/s12894-015-0049-y (PMC4465007; doi:10.1186/s12894-015-0049-y)
Supplement: Additional file 1: — Questions included in survey. [file 12894_2015_49_MOESM1_ESM.docx]

| Appendix 1 : Questions included in survey |
| --- |
| 1.Please state specialty |
| 2.Please state Hospital and Cancer Network |
| 3. Do you personally manage patients on active surveillance (AS) for prostate cancer?   - Yes - No |
| 4.Approximately how many men are placed on AS for prostate cancer in your unit in 1 year?   - Between 1-10 - Between 11-20 - Between 20-30 - >30 - Don’t know |
| 5. Where are men counselled about AS in your unit? (more than one answer possible)   - Urology prostate cancer clinic - Urology general clinic - Oncology clinic - Joint oncology and urology cancer clinic - Mixture of places/next available clinic space - Other (please specify) |
| 6. Who usually counsels men about AS in your unit? (more than one answer possible)  - Urologist  - Oncologist  - Joint clinic  - Nurse specialist  - Mixture of the above |
| 7. Do you have a unit/cancer network policy for selection of men suitable for AS?  - Yes  - No |
| 8. Which of the following do you use as criteria for AS selection? (more than one answer possible)  - Grade of 6  - Stage T2 or less  - PSA 10 or less  - PSA 10-20 with favourable characteristics  - Age  - Biopsy number of cores  - Biopsy % of core involvement |
| 9. Which of the following would you consider for AS ?    - T2A  - T2B  - T2C |
| 10. Which of the following would you consider NOT suitable for AS (more than one answer possible)  - Age > 75  - Age > 70  - Age < 60  - Age < 55  - Age < 50 |
| 11. Which of the following would you consider eligible for AS when considering number of cores involved?  - Biopsy cores < 10% involved  - Biopsy cores < 25% involved  - Biopsy cores < 50% involved  - Any involvement as long as Gleason 6 |
| 12. Which of the following would you consider eligible for AS when considering percentage of core involvement?  - <10% of total tissue involved  - <25% of total tissue involved  - <50% of total tissue involved  - Any involvement as long as Gleason 6 |
| 13. Do you use any of the following in your assessment of suitability for AS? (more than one answer possible)  - MRI of the prostate  - Bone scan (Tc99m)  - Template perineal biopsies  - Early repeat biopsies within 3 months  - Other |
| 14. Please state how you follow up AS patients with PSA Monitoring in the first 3 years  - 3 monthly  - 6 monthly  - 3 monthly for first 1-2 years then reducing frequency  - Other |
| 15. Please state how you follow up AS patients with their first repeat biopsy  - At 6 months  - At 12 months  - At 18 months  - Only re-biopsy if PSA rises or other clinical changes  - Other |
| 16. Please state how often you follow up AS patients with DRE  - 3 monthly  - 6 monthly  - 12 monthly  - Don’t do routine DRE |
| 17. Please state if you use imaging to follow up AS patients  - Yes  - No  - Patient choice  - MRI in patients who have initial suspicious lesion |
| 18. Who in your unit follows up men with AS (more than one answer possible)  - 2 clinician  - 1-2 clinicians  - more than 2 clinicians  - Specialist nurse  - mix of specialist nurse and clinicians |
| 19. Do you use the following for follow up of men on AS (more than one answer possible)  - PSA chart book  - Telephone follow up  - Other |
| 20. Where are men on AS followed up in your unit? (more than one answer possible)  - In a dedicated AS clinic  - In a dedicated prostate cancer clinic  - In a general clinician led clinic  - In a specialist nurse clinic  - A mixture of the above  - Telephone follow up and clinic visits when needed |
| 21. Do you give any additional lifestyle or dietary advice to patients that have selected AS?  - Lifestyle / Dietary advice-just verbally  - Lifestyle / Dietary advice generic leaflet  - Lifestyle / Dietary advice locally devised  - No  - Other |
| 22. Which of the following do you use as criteria to recommend conversion to active treatment (besides patient choice to convert)? (more than one answer possible)  - Upgrading on repeat biopsy  - Increased tumour volume  - Single rise in PSA velocity alone  - Two or more rises in PSA  - Rise in PSA prompts re biopsy first  - Change in DRE findings  - Changes in imaging findings  - Other |
